# Microorganisms in Superficial Deposits on the Stone Monuments in Saint Petersburg

**DOI:** 10.3390/microorganisms10020316

**Published:** 2022-01-29

**Authors:** Katerina V. Sazanova, Marina S. Zelenskaya, Alexey D. Vlasov, Svetlana Yu. Bobir, Kirill L. Yakkonen, Dmitry Yu. Vlasov

**Affiliations:** 1 Analytical Phytochemistry Laboratory, V.L. Komarov Botanical Research Institute of Russian Academy of Science, Professor Popov Street 2, 197376 St. Petersburg, Russia; 2 The Archive of the Russian Academy of Sciences, University Emb. 1, 199034 St. Petersburg, Russia; alex_vlasov@mail.ru; 3 Department of Botany, St. Petersburg State University, University Emb. 7/9, 199034 St. Petersburg, Russia; marsz@yandex.ru (M.S.Z.); dmitry.vlasov@mail.ru (D.Y.V.); 4 Department of Geology and Geoecology, Faculty of Geography, Herzen University, Moika Emb. 48, 191186 St. Petersburg, Russia; sv_bobir@mail.ru; 5 Department of Agricultural Chemistry, St. Petersburg State University, University Emb. 7/9, 199034 St. Petersburg, Russia; yakkonen@mail.ru; 6 Laboratory of Fungi Biochemistry, V.L. Komarov Botanical Research Institute of Russian Academy of Science, Professor. Popov Street 2, 197376 St. Petersburg, Russia

**Keywords:** superficial deposits, dark-colored fungi, organotrophic bacteria, cultural heritage, heavy metals

## Abstract

The composition of superficial deposits in urban environment and their importance in the development of the lithobiotic community of microorganisms has been investigated. Polyols, organic acids, mono- and disaccharides, as well as some amino acids, are the predominant low molecular weight organic components in superficial deposits, although the conditions on the stone surface are undoubtedly oligotrophic. Superficial deposits accumulate heavy metals, including Fe, Mn, Zn, Cu, Pb, and Cd, in surface sediments, among which the potentially toxic elements Zn, Cu, and Pb are accumulated in rather high concentrations. On model of *Aspergillus niger* as an example, it was shown micromycetes are resistant to heavy metals and retain their physiological activity when grown on this substrate. According to cultural studies, as well as metagenomic analysis, stress-resistant fungi and dark organotrophic bacteria are the main inhabitants of surface sediments. Probably, in the conditions of accumulation of superficial deposits on the stone, these organisms are the main inhabitants of the surface of the stone. With the development of more multi-species lithobiotic communities, they form the core of these communities. In the urban environment this type of primary colonization of the stone is likely realized.

## 1. Introduction

Historical monuments made of stone are an important component of the ubroecosystem of great cultural value. The problem of preserving of cultural heritage is especially important if the monuments are exhibited in the open air [1,2,3,4].

Cultural heritage stone monuments are colonized by different organisms. Lithobiotic communities include organisms of several large taxa of fungi, algae, bacteria, mosses, and lichens. Microbial communities at the stone–air interface are called subaerial biofilms (SABs). Subaerial biofilms can be viewed as multicomponent open ecosystems sensitively tuned to the atmosphere and the stone substratum [5]. Modern study indicates a complex structure of subaerial biofilm (SAB) formed on the surface of monuments of cultural heritage made of stone. Their research requires a variety of approaches, including studies of SAB functional features, taxonomic information, interactions with substrate, and studies of microbial responses to environmental stressors. The study of biofilms should be carried out at the systemic level, using “omic” approaches and mathematical models of their analysis [6].

Microbial community interacts with the underlying rock, which leads to additional weathering of stone and organomineral biodeposits formation on the stone surface, containing organic substances, products of bedrock weathering, as well as various elements getting from the environment (air, soil, etc.) in addition to the organisms themselves [7]. Lithobiotic organisms are indeed the main driving forces of biogeochemical cycles and provide the recycling of basic and trace elements [8]. Monuments may be degraded by growth and activity of living organisms. The study of the geochemical activity of the microbial community and its biochemical properties is an important problem for the preservation of cultural heritage [2,3,4,5,9].

**Figure 1:**
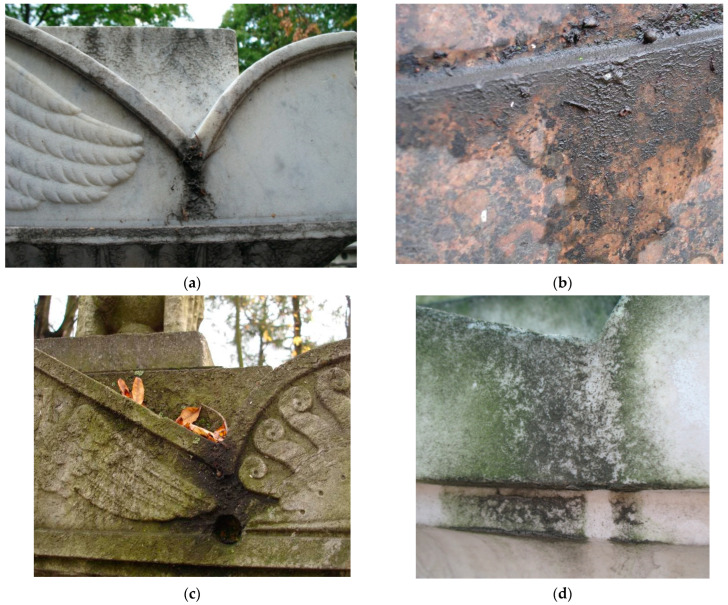
Superficial deposits on the surface of the monuments of the Historical Necropolises in Saint Petersburg: (**a**) Superficial deposits on marble monument; (**b**) superficial deposits on granite monument; (**c**) superficial deposits, leaf litter, and biofilms formation on the monument; (**d**) Algae biofilm growth on superficial deposits.

In urban ecosystems a significant part of SAB covering the stone surface is a black crust and superficial deposits that look like mud layers (Figure 1). Black crusts on the surface of the monuments were studied in various regions. Black crusts result from a sulfation reaction (SO_2_ reacts with calcite to form gypsum (CaSO_4_·2H_2_O)) and the entrapment of particles (especially soot), causing its blackening [10,11].

Traditionally, less attention has been paid to superficial deposits. Nevertheless, it is obvious that they accumulate various chemical contaminants coming from the environment, moisture, and organic matter from leaf litter (Figure 1c) which are potentially a medium for the development of microorganisms. Very often, superficial deposits coexist with biofilms of fungi, lichens, and algae (Figure 1d).

Presumably, superficial deposits can be considered as the primary type of layers on monuments or as the very initial stage of stone colonization. These layers are not only the primary source of nutrients for organisms, but also help to retain moisture and promote the adhesion of microorganisms on the stone, thus performing an environment-forming function for microorganisms. Thus, superficial deposits not only affect the state and appearance of the monument in themselves, but also play a very important role in the processes of its biodegradation. However, various elements, including heavy metals deposited from air aerosols, can also accumulate in these layers. The environmental conditions influence the extent of biofilm colonization and the biodeterioration processes. In large cities, the destruction of natural stone is noticeably accelerating, which is associated with the influence of an anthropogenic factor, primarily atmospheric pollution. They can affect the stone, as well as microorganisms, suppressing or stimulating their growth and changing their physiological properties. There are suggestions that in such habitats the aggressiveness of microorganisms increases, causing processes of biological damage to stone materials [9]. At the same time, it is known that a high concentration of heavy metals can have a toxic effect on microorganisms, suppressing their growth and physiological properties [12]. Despite the obvious role of superficial deposits in stone biodegradation, these statements are often generalized, and very little attention is paid to complex analytical studies of the chemical and biological composition of these deposits in specific regions. Since the ecological and climatic conditions of large cities are different, case studies are of great importance in specific regions.

The aim of this work is to describe the biological communities and chemical composition of superficial deposits on the surface of stone monuments in Saint Petersburg and evaluate the role of primary layers as a habitat for organisms and a factor of the destruction of stone monuments

## 2. Materials and Methods

### 2.1. Sampling

The deposits samples were collected in Saint Petersburg from the surface of granite and marble monuments of the Museum Necropolis of the 18th century, which is located in a small area in the central part of Saint Petersburg. Samples were collected from horizontal, vertical, and inclined rock surfaces. 37 samples from the surface of 37 tombstones were collected for micromycetes identification: 20 samples from carbonate rocks (marble, limestone) and 17 samples from silicate rocks (Rapakivi, Serdobolsky granites). Analysis of each sample for species composition was carried out by direct inoculation of particles of superficial deposits on the nutrient medium in Petri dishes. The abundance of certain species in each particular of Petri dishes was not taken into account. The frequency of occurrence of species (how many times the species of fungus was detected in 37 samples, in%) was analyzed.

### 2.2. Microorganism Identification

#### 2.2.1. Cultural Studies of Micromycetes

For identification of micromycetes, small fragments of superficial deposits washings (by distilled water sterilized by autoclaving) were placed on the surface Czapek-Dox nutrient medium (HiMedia, Mumbai, India) and incubated for 2–4 weeks at a temperature of 25 °C. Identification was carried out in accordance with morphological characteristics using guidebooks [13,14,15,16] and verified by Index Fungorum electronic database [17].

#### 2.2.2. Metagenomic Analysis

Commercial PowerSoil DNA Isolation kit (MO BIO Laboratories, Inc, Carlsbad, CA, USA) and protocols described in the literature [18] were used to isolate DNA. Metagenomic analysis was used to determine a wide range of bacteria and micromycetes in primary layers sampled from the surface of monuments. Bacteria were identified based on the analysis of 16S rRNA genes. Deep sequencing of the 16S RNA gene was performed using the IonTorrentPGM system (LifeTechnologies, Carlsbad, CA, USA). A library for sequencing the 16S-RNA gene was prepared by amplifying the V4-V5 region [19] using primers U515F (5′-GTGCCAGCMGCCGCGGTAA-3′) [20] and 926R (5′-CCGTCAATTCMTTTRAGT-3′) [21]. Amplification was carried out using the FastStartHighFidelityPCRsystem kit (Roche, Basel, Switzerland). Analysis of diversity was performed using the Qiime software package (version 1.9.0) according to the standard protocol. When forming the OTU, a sequence similarity threshold of 97% was used. The classification was carried out on the basis of comparison with the Greengenes database.

Identification of micromycetes in biofilms was carried out using primers for amplification of the site (ITS1—5.8 S—ITS2) (Beagle, St Petersburg, Russia):

ITS4 TCCTCCGCTTATTGATATGC

gITS7 GTGARTCATCGARTCTTTG

Sequencing was performed using the IonTorrentPGM system (LifeTechnologies). Filtration quality—standard > Q20. Part of the work related to the analysis was carried out using the Qiime protocol [22]. Primers were removed from the reads, reads without primers were removed from the analysis, the remaining reads were cut to the same length of maximum quality, the data obtained were processed using the DADA2 protocol to detect exact variants sequences [23]. Species identification was carried out using the full match algorithm in DADA2 using the sequences of the SILVA database [24].

### 2.3. Analysis of Low-Molecular Weight Substances

For the analysis of small organic molecules, 10 samples were taken. Three of them were rich in leaf litter of *Sorbus* sp., three samples leaf litter of Betula sp., and for four more samples it is impossible to speak about the predominance of leaves of any tree. Samples of superficial deposits were extracted twice by methanol (Vecton, St Petersburg, Russia). Extracts were combined and centrifuged (10 min, 400× *g*) at room temperature, dried by a rotary evaporator at 40 C and soluble in pyridine (30 μL). For derivatization BSTFA (N,O-bis—3-methyl-silyl-3-F-acetamide) (30 μL) was used (incubated at 100 °C for 15 min). The samples were analyzed by gas chromatography–mass spectrometry (GC-MS) on a Maestro instrument (Interlab, Moscow, Russia) with an Agilent 5975 mass-selective detector (Agilent, Santa Clara, CA, USA). Column HP-5MS, 30 m × 0.25 mm × 0.25 μm. Chromatography was performed with temperature from 70 to 320 °C for 6 °C/min in the mode of constant carrier gas flow through the column (1 mL/min). Helium (purity 99.9999%, Linde, Moscow, Russia) was used as the carrier gas. Chromatograms were recorded using the total ion current. Mass spectra were scanned in the range of 50–750 *m*/*z* with a frequency of 1.6 scans/s. For processing of mass spectrometric information AMDIS program (2.65 version) [25], the standard NIST2005 library, and the library of standard compounds of BIN RAS were used. Quantitative analysis was carried out by an internal standard method using tridecane in the UniChrom program (5.0.19.1180 version) [26].

### 2.4. Analysis of Heavy Metals

Samples of superficial deposits were digested in concentrated HNO_3_ (NevaReaktiv, St Petersburg, Russia) in a microwave digestion system Minotavr-2 (Lumex, St Petersburg, Russia). Concentrations of heavy metals were determined by electrothermal atomic absorption spectrometry (AAS) MGA 915 (Lumex, St Petersburg, Russia) with Zeeman-effect background correction.

### 2.5. Experimental Study

To observe the development of microscopic fungi in superficial deposits in vitro fragments of the layer from the stone surface were used. Fragments of superficial (approximately 1 g) deposits collected from the marble surface of monuments in Necropolis were autoclaved and put on the bottom of plastic Petri dishes and 15 mL of Czapek-Dox liquid medium (NaNO_3_—3.0; KH_2_PO_4_—1.0; MgSO_4_·7H_2_O—0.5; KCl—0.5; FeSO_4_·7H_2_O—0.015; glucose—30.0 g/L) was added. Aspergillus niger (strain Ch4/07; GenBank accession no-KF768341) was inoculated by conidia and mycelium fragments. Inoculum culture was cultivated 10 day on Czapek-Dox agar medium.

Observations were carried out within 14 days using light microscopy and scanning electron microscopy (SEM).

### 2.6. Scanning Electron Microscopy and Energy-Dispersive X-ray (EDX) Spectroscopy

SEM with EDX analysis was carried out by using a TM 3000 (HITACHI, Tokyo, Japan, 2010) with OXFORD EDX module and Oxford Inca system for EDX measurements. Oxford Inca system operated in a low vacuum (60 Pa) mode and at an acceleration voltage of 15 kV.

### 2.7. Statistical Analysis

For calculation the Averages with standard deviation Microsoft Excel 2016 software were used.

## 3. Results

### 3.1. Taxonomic Composition of Microorganisms in Superficial Deposits on Stone Monuments

#### 3.1.1. Cultural Studies

As a result of mycological studies using cultural methods, 37 species of micromycetes were identified in the superficial deposits of the monuments of the Museum necropolises (Table 1). The dominant species were dark-colored fungi: *Alternaria alternata*, *Cladosporium cladosporioides*, *Coniosporium* sp., *Phoma herbarum*. The absolute dominant in frequency of occurrence was fungus *Aureobasidium pullulans*.

#### 3.1.2. Metagenomic Study

As a result of metagenomic analysis (Table 2), a rather large number of basidiomycetes, which cannot be determined by cultural methods on Czapek-Dox medium, were revealed in the superficial deposits. The number of major taxonomic groups was: *Ascomycetes*—87.8%; *Basidiomycetes*—7.7%; *Glomeromycetes*—0.2%, and *Chytridiomycetes*—0.6%. Representatives of the genera *Aureobasidium* (13.8%) and *Celosporium* (10.3%) had the highest percentage of occurrence. At least five genera *Aureobasidium*, *Capnobotryella*, *Exophiala*, *Endoconidioma*, *Celosporium* (total 32%) include melanized dark-colored fungi.

As a result of metagenomic analysis of bacteria (Table 3), representatives of 17 bacterial phyla were identified. The results obtained indicate that bacteria from the two main groups *Bacteroidetes* and *Proteobacteria* predominate in the superficial deposits on stone monuments. *Actinobacteria* and *Acidobacteria* constitute a significant part of the community (7.5 and 6.6%, respectively).

At the genus level, more than 90 taxa of bacteria have been identified, most of which are organotrophic bacteria characteristic of soil habitats. The most significant genera (proportion at least 1%) are presented in Table 4.

### 3.2. Low Molecular Weight Composition of Superficial Deposites

In the samples from the surface of the granite and marble monument sugar alcohols, sugars, amino acids, organic acids were found. About 30 different compounds, including some unidentified ones, were detected. The quality composition of low molecular weight compounds was presented in Table 5. The quantitative composition of compounds in layers with leaf litter particles was specific. The layers with leaf litter of *Betula* sp. were dominated by glycerol, phosphate, malic acid, ribose, and glucose (maximum). In leaf litter of *Sorbus* sp., the sugar content was more than five times lower than in litter of *Betula* sp., but the sugar alcohols arabitol and sorbitol predominated.

The total amount of sugar and polyols ranged from 3 to 5 mg/g dry weight in different samples. The total amount of amino acids was 1–3 µ/g of dry weight.

### 3.3. Heavy Metals Composition in Superficial Deposites

By AAS in the samples of superficial deposits, the heavy metals including Fe, Mn, Zn, Cu, Pb, and Cd were detected (Table 6). Fe was found in the highest concentration. Cd was detected in the smallest amount; the differences between Cd concentrations in the samples reached 10 times.

### 3.4. Aspergillus Niger Interactions with Superficial Deposits In Vitro

**Figure 2:**
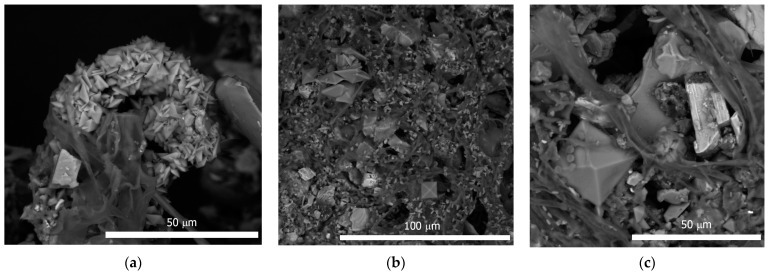
*A. niger* grown on superficial deposit in the experiment: (**a**) Ca-oxalates clusters on *A. niger* mycelium; (**b**) formation a lot of Ca-oxalates crystals and accumulation of minerals particles on *A. niger* mycelium; (**c**) weddellite crystal and minerals particles on *A. niger* mycelium.

**Figure 3:**
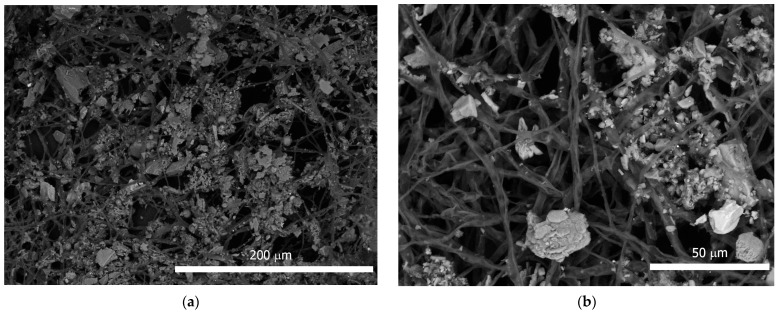

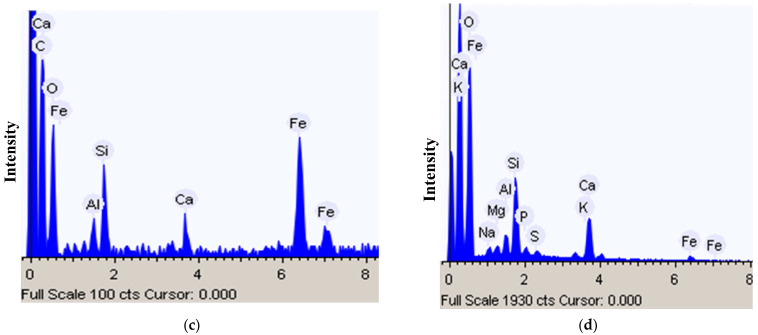
Minerals particles on *A. niger* mycelium: (**a**,**b**) Particles adhesion on mycelium; (**c**,**d**) EDX spectrum of mineral particles.

*A. niger* was actively growing in a surface culture on a nutrient medium with a fragment of the superficial deposit, forming a well-developed mycelium and abundant sporulation. A characteristic phenomenon observed in this experiment was the formation of crystals of weddellite calcium oxalate dihydrate in the mycelium; the size of the crystals varied from 3 to 25 µm (Figure 2). In some cases, calcium oxalates formed clumps and aggregates (Figure 3a).

In addition, intense adhesion of mineral grains and granules was observed in the mycelium (Figure 2c and Figure 3a,b). EDX analysis showed the predominance of Fe, Ca, Si, and Al among the mineral elements of these particles (Figure 3c,d). In the EDX spectrum, Na, K, and Mg were also present, the presence of which was probably due to the nutrient medium.

## 4. Discussion

According to the data obtained, superficial deposits on stone monuments represent a specific habitat for a diverse community of microorganisms, especially for extremotolerant species of fungi and bacteria. The dominance of dark-colored micromycetes was confirmed by both cultural and molecular methods. So-called group black fungi include yeasts as well as meristematic fungi. Taxonomically, they represent a wide and heterogeneous group of black pigmented fungi that share, as common characteristic, the presence of melanins within the cells (swollen cells, hyphae and/or spores). The production of melanin and the meristematic development allow them to survive in stressed environmental conditions such as low humidity and high sun irradiation [27]. For this group of fungi, the term of rock-inhabiting fungi is also used to underline the exclusive isolation of many of them from rock surfaces [28]. Dark-colored fungi are adapted to unfavorable and extreme habitat conditions which are characteristic of the surface of the stone [29,30]. Moreover, these fungi can grow with only a limited supply of carbon, which might be contained in dust, microbial products, or pollutants [31,32,33].

The core of the microbial community—the dominant and frequently occurring species—correspond to those in subaerial biofilms and primary soil [34]. Apparently, the dark-colored species of fungi that dominate in the superficial deposits are the primary inhabitants of monuments in the urban environment. Further, with the development of more complex and diverse multispecies communities, these species form the core of the mycobiota. Likely this type of layering can be regarded as the primary stage of stone colonization and the initial stage of the formation of communities.

Among the identified bacteria, there are species of extreme habitats (psychrophilic conditions, places with high level of chemical pollution, etc.). At the same time, the share of participation of cyanobacteria in surface layers was quite low (only 2%). This fact suggests that the formation of microbiota in surface layers does not proceed in the same way as on an open stone, where phototrophic microorganisms are pioneers of occlusion of the stone surface.

The growth of heterotrophic bacteria and fungi on stone is needed by metabolites of autotrophic organisms or organic substances coming from the environment [3,35]. Probably in the case of accumulation of surface deposits on the stone, they are the source of nutrients for the development of heterotrophic organisms. The predominant low-molecular-weight organic components in the superficial deposits on monuments are sugar alcohols, organic acids, mono-and disaccharides. Some amino acids were also detected. These compounds are readily assimilated by fungi and bacteria. The total amount of potential low-molecular carbon sources for the fungi varied from 3 to 5 mg/g of dry matter in different samples. The total amount of amino acids is 1–3 µ/g of substrate. These indices that conditions in the superficial deposits on the stone surface are undoubtedly oligotrophic, but still maintain a number of nutrient sources which is quite sufficient for fungi of this ecological group.

In general, the qualitative composition of low-molecular-weight organic components in superficial deposits is much poorer than the composition of biofouling and primary soils [34]. Phenolic compounds, terpenoids, and sterols, which are typical components of the metabolome of many living organisms, were not found in the composition of superficial deposits.

Most likely, the main share of the detected organic matter is not so much the microorganisms themselves present in the mud layer as exogenous sources, for example, leaf litter. Additionally, leaf litter accumulation on the monuments may explain the presence of basidiomycetes, as well as micromycetes, usually mentioned in connection with plant substrates (phytopathogens or saprotrophs on plant residues). Their findings are probably due to the ingress of fungal propagules from the surrounding plants onto the stony substrate.

According to a number of authors, air pollution in cities is one of the most significant factors affecting stone buildings and structures, as well as cultural heritage sites [36,37]. Our results showed accumulation of heavy metals including Fe, Mn, Zn, Cu, Pb, and Cd in superficial deposits, among which the potentially toxic elements Zn, Cu, and Pb have been accumulated in fairly high concentrations. However, dark-colored micromycetes and extremotolerant bacteria generally exhibit resistance to heavy metals.

In experimental conditions, the presence of a sample of superficial deposits in nutrient medium has not suppressed the growth of mycelium of *A. niger*. *A. niger* showed a pronounced ability to adsorb mineral particles on mycelium. Among heavy metals, the concentration of Fe in the samples of the layers was two orders of magnitude (or more) higher than the concentration of other heavy metals, which probably explains its predominance in the EDX spectra of particles adhered to mycelium.

As shown by the results of our previous study [38] many elements, including Pb and Fe, unevenly cover the surface of the monument, but are present in the composition of mineral grains, sometimes distributed over the surface. The fungi are likely characterized by the adhesion of large particles on mycelium, among which Fe-containing grains clearly predominate.

In samples inoculated with *A. niger*, formation of calcium oxalate crystals is observed. The ability of *A. niger* to release oxalic acid and participate in biogenic crystallization of metal oxalates is well described [39,40,41,42,43].

Our experiments have shown that superficial deposits are actively involved in biogeochemical interactions. Their composition promotes the formation of calcium oxalates. No other metal oxalates were found. Probably, due to the excess calcium content in the composition of superficial deposits, oxalic acid was bound with calcium. In the initial samples before the inoculation of *A. niger*, no calcium oxalates were found, which is probably due to the low activity of the microbial community.

## 5. Conclusions

The main inhabitants of superficial deposits are dark-colored stress-resistant fungi and organotrophic bacteria. Superficial deposits contain a relatively small amount of microorganisms. At the same time, they are a source of nutrients for the growth of the lithobiotic community and obviously contribute to its development on stone. Superficial deposits are highly bioreceptive, easily adhered by growing mycelium, and interact with the metabolic products of *A. niger*. In urban conditions, when surface deposits are intensively accumulating on the stone, dark-colored fungi are probably the main inhabitants of the stone surface. Further, with the development of more complex and diverse multispecies lithobiotic communities, these species form the core of the mycobiota. Our data suggest that in the urban environment, this type of primary colonization of the stone is realized.

## Figures and Tables

**Table 1:** Micromycetes identified in superficial deposits by cultural methods.

Species	Frequency of Occurrence, %
*Alternaria alternata* (Fr.) Keissl.	38
*Ascochyta* sp.	3
*Aspergillus flavus* Link	3
*Aspergillus niger* Tiegh.	16
*Aspergillus ochraceus* Wilh.	3
*Aspergillus reptans* Samson and W. Gams 1986	8
*Aspergillus versicolor* (Vuill.) Tirab.	3
*Aureobasidium pullulans* (de Bary) G. Arnaud	57
*Botrytis cinerea* Pers	8
*Cladosporium cladosporioides* (Fresen.) G.A. de Vries	30
*Cladosporium herbarum* (Pers.) Link	3
*Coniosporium* sp.	19
*Epicoccum nigrum* Link	11
*Fusarium oxysporum* Schltdl.	11
*Fusarium* sp.	5
*Mortierella lignicola* (G.W. Martin) W. Gams and R. Moreau	3
*Papulaspora* sp.	8
*Penicillium brevicompactum* Dierckx	16
*Penicillium citrinum* Thom	3
*Penicillium* sp.	11
*Penicillium herquei* Bainier and Sartory	3
*Phaeosclera dematioides* Sigler, Tsuneda and J.W. Carmich.	5
*Didymella glomerata* (Corda) Qian Chen and L. Cai	5
*Phoma herbarum* Westend.	22
*Phoma* sp.	3
*Pythium* sp.	3
*Sarocladium strictum* (W. Gams) Summerb.	14
*Sclerotinia sclerotiorum* (Lib.) de Bary	3
*Scytalidium lignicola* Pesante	14
*Sydowia polyspora* (Bref. and Tavel) E. Müll.	8
*Taenionella* sp.	3
*Talaromyces purpureogenus* Samson, Yilmaz, Houbraken, Spierenb., Seifert, Peterson, Varga and Frisvad	3
*Trichocladium asperum* Harz	3
*Trichoderma viride* Pers.	11
*Alternaria chartarum* Preuss	8

**Table 2:** Micromycetes identified in superficial deposits (according to the results of metagenomic analysis).

Genus	Proportion, %
*Aureobasidium* (*Botryosphaeriaceae*)	13.8
*Capnobotryella* (*Capnodiales*)	0.2
*Endoconidioma* (*Dothideaceae*)	2.3
*Celosporium* (*Dothideaceae*)	10.3
*Phoma* (*Pleosporales*)	0.2
*Pyrenochaeta* (*Pleosporales*)	0.6
*Lophiostoma* (*Pleosporales*)	0.5
*Unidentified genera* (*Pleosporales*)	9.2
*Capronia* (*Chaetothyriales*)	0.6
*Exophiala* (*Chaetothyriales*)	5.5
*Phialosimplex* (*Eurotiales*)	0.9
*Sarcinomyces* (*Eurotiales*)	0.4
*Phialocephala* (*Helotiales*)	0.8
*Geomyces* (*Incertae sedis*)	0.6
*Dactylella* (*Orbiliales*)	0.9
*Ascomycota*, *unidentified*	22.7
*Agaricomycetes*, *Auriculariales*, *unidentified*;	6.1
*Sporobolomyces* (*Basidiomycota*, *Microbotryomycetes*)	0.2
*Guehomyces* (*Basidiomycota*, *Tremellomycetes*)	0.3
*Powellomyces* (*Chytridiomycetes*)	0.5
*Glomeromycetes*, *unidentified*	0.2
*Mortierella* (*Zygomycota*)	0.2
Lichen genus (mycobiont)	
*Solenopsora* (*Lecanorales*)	6.7
*Lecanora* (*Lecanorales*)	0.2
*Oropogon* (*Lecanorales*)	0.3
*Phaeophyscia* (*Teloschistales*)	0.4
*Caloplaca* (*Teloschistales*)	0.2

**Table 3:** The main taxonomic groups of bacteria in superficial deposits (according to the results of metagenomic analysis).

Taxonomy	Proportion, %
*Acidobacteria*	6.6
*Actinobacteria*	7.5
*Armatimonadetes*	0.8
*Bacteroidetes*	40.5
*Chlamydiae*	0.0
*Chlorobi*	0.1
*Chloroflexi*	0.4
*Cyanobacteria*	1.7
*FBP*	3.3
*Fibrobacteres*	0.0
*Firmicutes*	0.0
*Gemmatimonadetes*	0.1
*OD1*	0.1
*Planctomycetes*	1.1
*Proteobacteria*	33.4
*TM7*	0.4
*Verrucomicrobia*	0.1
*Thermi*	3.6

**Table 4:** Some bacteria taxa (genus level) characterized the microbiota (according to the results of metagenomic analysis).

Taxonomy	Proportion, %
*Friedmanniella*	2.1
*Cytophaga*	2.2
*Hymenobacter*	5.2
*Spirosoma*	3.7
*Flavobacterium*	8.9
Pedobacter	3.4
*Chitinophagaceae*	6.4
Cyanobacteria	2.0
*Rubellimicrobium*	1.9
*Sphingomonas*	2.1
*Oxalobacteraceae*	1.2
*Deinococcus*	3.7

**Table 5:** The quality composition of low molecular weight compounds in superficial deposits.

Organic Acids	Fatty Acids	Amino Acids	Sugars	Polyols	Other Compounds
Succinic acid Glyceric acid Fumaric acid Citric acid Malic acid Erythronic acid	aC 16.0 aC 22.0 aC 26.0	Alanine Glycine Serine Threonine Proline	Glucose aP Glucose bP Fructose aF Fructose bF Galactose F Ribose Sucrose Maltose Arabinose	Erythritol Arabitol Mannitol Chiro-inositol Myo-inositol	Phosphate Uridine

**Table 6:** Content of metals in superficial biodeposits.

Metal	Concentration of Metals *, μg/g
Fe	32,280.6 ± 3009.0
Mn	414.7 ± 43.4
Zn	502.2 ± 26.3
Cu	500.9 ± 18.4
Pb	122.9 ± 6.4
Cd	8.5 ± 9.8

* Averages and standard errors of heavy metals.

## References

[1] De Leo F., Urzi C., Misra J.K., Tewari J.P., Deshmukh S.K., Vágvölgyi C. (2015). Microfungi from deteriorated materials of cultural heritage. Fungi from Different Substrates.

[2] Kurakov A.V., Somova N.G., Ivanovskii R.N. (1999). Micromycetes populating limestone and red brick surfaces of the Novodevichii convent masonry. Microbiology.

[3] Warscheid T., Braams J. (2000). Biodeterioration of stone: A review. Int. Biodeterior. Biodegrad..

[4] Salvadori O., Municchia A.C. (2015). The role of fungi and lichens in the biodeterioration of stone monuments. Open Conf. Proc. J..

[5] Gorbushina A. (2007). Life on the rocks. Environ. Microbiol..

[6] Villa F., Stewart P.S., Klapper I., Jacob J.M., Cappitelli F. (2016). Subaerial Biofilms on Outdoor Stone Monuments: Changing the Perspective Toward an Ecological Framework. BioScience.

[7] Martino P.D. (2016). What About Biofilms on the Surface of Stone Monuments?. Open Conf. Proc. J..

[8] Rousk J., Bengtson P. (2014). Microbial regulation of global biogeochemical cycles. Front. Microbiol..

[9] Scheerer S., Ortega-Morales O., Gaylarde C. (2009). Chapter 5 Microbial Deterioration of Stone Monuments—An Updated Overview. Adv. Appl. Microbiol..

[10] Comite V., Ricca M., Ruffolo S.A., Graziano S.F., Rovella N., Rispoli C., Gallo C., Randazzo L., Barca D., Cappelletti P. (2020). Multidisciplinary Approach for Evaluating the Geochemical Degradation of Building Stone Related to Pollution Sources in the Historical Center of Naples (Italy). Appl. Sci..

[11] Farkas O., Siegesmund S., Licha T. (2018). Geochemical and mineralogical composition of black weathering crusts on limestones from seven different European countries. Environ. Earth Sci..

[12] Siddiquee S., Rovina K., Azad S., Naher L., Suryani S., Chaikaew P. (2015). Heavy Metal Contaminants Removal from Wastewater Using the Potential Filamentous Fungi Biomass: A Review. J. MicrobBiochem. Technol..

[13] Ellis M.B. (1971). Dematiaceous Hyphomycetes.

[14] Ellis M.B. (1976). More Dematiaceous Hyphomycetes.

[15] De Hoog G.S., Guarro J. (1995). Atlas of Clinical Fungi.

[16] De Hoog G.S., Hermanides-Nijhof E.J. (1977). Survey of the black yeasts and allied fungi. Stud. Mycol..

[17] Index Fungorum. http://www.indexfungorum.org.

[18] Vladimirov I.A., Matveeva T.V., Lutova L.A. (2014). Real-Time PCR to Study the Distribution of Agrobacteria.

[19] Fujimoto M., Moyerbrailean G.A., Noman S., Gizicki J.P., Ram M.L., Green P.A. (2014). Application of Ion Torrent Sequencing to the Assessment of the Effect of Alkali Ballast Water Treatment on Microbial Community Diversity. PLoS ONE.

[20] Turner S., Pryer K.M., Miao V.P., Palmer J.D. (1999). Investigating Deep Phylogenetic Relationships among Cyanobacteria and Plastids by Small Subunit rRNA Sequence Analysis. J. Eukaryot. Microbiol..

[21] Haas B.J., Gevers D., Earl A.M., Feldgarden M., Ward D.V., Giannoukos G., Ciulla D., Tabbaa D., Highlander S.K., Sodergren E. (2011). Chimeric 16S rRNA sequence formation and detection in Sanger and 454-pyrosequenced PCR amplicons. Genome Res..

[22] Qiime. http://qiime.org/index.html.

[23] Callahan B.J., McMurdie P.J., Rosen M.J., Han A.W., Johnson A.J. (2016). Holmes, S.P. DADA2: High-resolution sample inference from Illumina amplicon data. Nat. Methods..

[24] Quast C., Pruesse E., Yilmaz P. (2013). The SILVA ribosomal RNA gene database project: Improved data processing and web-based tools. Nucleic Acids Res..

[25] AMDIS. http://www.amdis.net/index.html.

[26] UniChrom. http://www.unichrom.com/unichrome.shtml.

[27] De Hoog G.S. (1993). Evolution of black yeasts: Possible adaptation to the human host. Antonie Van Leeuwenhoek.

[28] De Leo F., Urzì C. (2003). Fungal colonization on treated and untreated stone surfaces. Molecular Biology and Cultural Heritage.

[29] Sterflinger K., Tesei D., Zakharova K. (2012). Fungi in hot and cold deserts with particular reference to microcolonial fungi. Fungal Ecol..

[30] Gorbushina A.A., Whitehead K., Dornieden T., Niesse A., Schulte A., Hedges J.I. (2003). Black fungal colonies as unit of survival: Hyphal mycosporines synthesized by rock dwelling microcolonial fungi. Can. J. Bot..

[31] Saiz-Jimenez C. (1997). Biodeterioration vs. biodegradation: The role of microorganisms in the removal of pollutants deposited onto historic buildings. Int. Biodeterior. Biodegrad..

[32] Viles H.A., Gorbushina A.A. (2003). Soiling and microbial colonisation on urban roadside limestone: A three year study in Oxford, England. Build. Environ..

[33] Moroni B., Pitzurra L. (2008). Biodegradation of atmospheric pollutants by fungi: A crucial point in the corrosion of carbonate building stone. Int. Biodeterior. Biodegrad..

[34] Sazanova K.V., Zelenskaya M.S., Rodina O.A., Shavarda A.L., Vlasov D.Y. (2021). Metabolomic Profiling of Biolayers on the Surface of Marble in Nature and Urban Environment. Case Study of Karelia and St. Petersburg. Minerals.

[35] Hoffland E., Kuyper T.W., Wallander H., Plassard C., Gorbushina A.A., Haselwandter K., Holmström S., Landeweert R., Lundström U.S., Rosling A. (2004). The role of fungi in weathering. Front. Ecol. Environ..

[36] Jönsson A. (2011). Ni, Cu, Zn, Cd and Pb in Sediments in the City-Center of Stockholm, Sweden. Origins, Deposition Rates and Bioavailability.

[37] Fernandez-Camacho R., Rodrigues S. (2012). Ultrafine particle and fine trace metal (As, Cd, Cu, Pb and Zn) pollution episodes induced by industrial emissions in Huelva, SW Spain. Atmos. Environ..

[38] Sazanova K.V., Zelenskaya M.S., Manurtdinova V.V., Izatulina A.R., Rusakov A.V., Vlasov D.Y., Frank-Kamenetskaya O.V. (2021). Accumulation of Elements in Biodeposits on the Stone Surface in Urban Environment. Case Studies from Saint Petersburg, Russia. Microorganisms.

[39] Gadd G.M. (1999). Fungal Production of Citric and Oxalic Acid: Importance in Metal Speciation, Physiology and Biogeochemical Processes. Adv. Microb. Physiol..

[40] Sayer J.A., Gadd G.M. (2001). Binding of cobalt and zinc by organic acids and culture filtrates of Aspergillus niger grown in the absence or presence of insoluble cobalt or zinc phosphate. Mycol. Res..

[41] Gadd G.M. (2007). Geomycology: Biogeochemical transformations of rocks, minerals, metals and radionuclides by fungi, bioweathering and bioremediation. Mycol. Res..

[42] Sazanova K.V., Vlasov D.Y., Osmolovskay N.G., Schiparev S.M., Rusakov A.V., Frank-Kamenetskaya O.V., Panova E.G., Vlasov D.Y. (2016). Significance and regulation of acids production by rock-inhabited fungi. Biogenic-Abiogenic Interactions in Natural and Anthropogenic Systems.

[43] Sazanova K.V., Frank-Kamenetskaya O.V., Vlasov D.Y., Zelenskaya M.S., Vlasov A.D., Rusakov A.V., Petrova M.A. (2020). Carbonate and Oxalate Crystallization by Interaction of Calcite Marble with *Bacillus subtilis* and *Bacillus subtilis–Aspergillus niger* Association. Crystals.

